# Osteoporosis documentation following hip fracture: a retrospective cohort study from a tertiary hospital

**DOI:** 10.1186/s12891-026-09745-6

**Published:** 2026-03-23

**Authors:** Yehudit Eden-Friedman, Alla Lizeachin, Slava Gezunterman, Alon Friedlander, Bernice Oberman, Liat Toderis, Iris Reychav, Iris Vered, Liana Tripto-Shkolnik

**Affiliations:** 1 Division of Endocrinology, Diabetes and Metabolism, Sheba Medical Center, Tel Hashomer, Ramat Gan, Israel; 2 Gray Faculty of Medical and Health Sciences, Tel Aviv University, Tel Aviv, Israel; 3 Department of Orthopedic Surgery, Sheba Medical Center, Tel Hashomer, Ramat Gan, Israel; 4 ADAMS Center, Sheba Medical Center, Tel Hashomer, Ramat Gan, Israel; 5 Industrial Engineering and Management Department, Ariel University, Derech Sheba 2, Ramat Gan, 5262000 Israel

**Keywords:** Osteoporosis, Hip fracture, Documentation, Diagnosis recording, Fracture liaison service, Nurse practitioner

## Abstract

**Background:**

Hip fractures are among the most common consequences of osteoporosis, yet adequate diagnosis in medical records remains suboptimal, and translation of documented osteoporosis into pharmacologic therapy is often incomplete. This study evaluated whether integration of a dedicated nurse practitioner into the orthopedic inpatient workflow was associated with improved osteoporosis documentation following hip fracture. A secondary, exploratory aim was to examine associations between documentation and downstream clinical outcomes.

**Methods:**

Data were extracted from the MDClone big data platform (ADAMS), and included patients aged ≥ 60 hospitalized with ICD-10 coded hip fractures between 2007-2024.

**Results:**

The cohort comprised 6,933 patients, of whom 4,405 (63.5%) were women, with a mean age of 81.3 years. A total of 4,150 patients (59.8%) were discharged with a diagnosis of osteoporosis or osteoporotic fracture. A sustained increase in documentation rates was observed after 2015, coinciding with the integration of a nurse practitioner into inpatient care. Recorded use of anti-osteoporosis pharmacologic therapy was low and similar between groups. No consistent differences were observed in secondary clinical outcomes.

**Conclusions:**

The rate of osteoporosis documentation following hip fractures was substantially higher than reported in the literature from centers without fracture liaison services. Integration of a dedicated nurse practitioner into the orthopedic inpatient workflow was associated with a significant and sustained improvement in osteoporosis documentation. These findings highlight the potential of targeted inpatient interventions to improve post-fracture osteoporosis recognition and documentation.

**Trial registration:**

Not applicable. This is a retrospective observational study.

**Supplementary Information:**

The online version contains supplementary material available at 10.1186/s12891-026-09745-6.

## Background

A low-trauma hip fracture in postmenopausal women or in men aged ≥ 50 years is sufficient to establish a diagnosis of osteoporosis, irrespective of bone mineral density (BMD) measurements [1, 2]. Despite this, the documentation of osteoporosis following hip fractures remains suboptimal, with various studies reporting diagnosis rates as low as about 30% [3–6]. Notably, a formal diagnosis of osteoporosis increases the likelihood of initiating pharmacological treatment [7–10]. Nevertheless, even when the diagnosis is appropriately made, many patients still fail to receive adequate medical management [7, 11–13].

Fracture Liaison Services (FLS) provide structured post-fracture care aimed at reducing the risk of future fractures through pharmacological intervention, lifestyle modifications and rehabilitation service. Numerous studies [14] have demonstrated the utility of FLS, showing improved rates of osteoporosis medication initiation and a reduction in subsequent fractures. Implementation of FLS recommendations may be associated with reduced mortality [15], although causality remains uncertain due to methodological limitations in existing studies.

FLS may not be feasible in all settings due to logistical and financial constraints. Consequently, simpler alternative strategies for post-fracture care and follow-up have been explored, often with favorable outcomes. These include electronic medical record (EMR) alerts [16], orthogeriatric co-management [17], pharmacist-led interventions [18], and primary care–driven follow-up [19]. In some healthcare systems, virtual FLS models [10, 20] have also emerged as solutions for coordinated osteoporosis management. At our institution, Sheba Medical Center, a formal FLS is not currently in place. However, in 2014, a dedicated nurse practitioner was incorporated into the orthopedic ward team to facilitate appropriate post-fracture care for patients with hip fractures.

The primary aim of this study was to evaluate changes in the rate of osteoporosis documentation following hip fractures, particularly in relation to the integration of a dedicated nurse practitioner into the orthopedic inpatient workflow. Secondary, hypothesis-generating analyses explored whether the presence of a documented osteoporosis diagnosis was associated with long-term outcomes, including all-cause mortality, subsequent fractures, rehospitalizations, and osteoporosis medication use.

## Materials and methods

We conducted a retrospective cohort study of all patients aged ≥ 60 years who were hospitalized at Sheba Medical Center following a hip fracture between 2007 and 2024. The study was approved by the Institutional Review Board (IRB) of Sheba Medical Center, Israel (approval #1989-25-SMC-D), and was conducted in accordance with the principles of the Declaration of Helsinki, with a waiver of informed consent.

Data extraction was conducted using the MDClone ADAMS big-data platform (MDClone, 2025) [21], a rigorously validated software system designed for accurate and efficient retrieval of clinical information. The platform applies standardized event definitions and precise temporal mapping, ensuring methodologically consistent cohort construction and high reproducibility across queries [22–24]. During the study period, diagnostic coding practices in Israel transitioned from ICD-9 to ICD-10. Within the MDClone platform, historical ICD-9 codes were systematically mapped to ICD-10 codes, allowing uniform identification of hip fracture diagnoses across the entire study period. Under IRB approval, patient-level data were obtained for all individuals with ICD-10–coded hip fractures, including femoral neck fractures (Garden stages 1–4), intertrochanteric, pertrochanteric, subtrochanteric, greater trochanter, femoral head, and other proximal femur fracture sites.

A complete list of included diagnoses is provided in Supplement A. Patients with isolated subtrochanteric fractures, periprosthetic fractures, or fractures resulting from major trauma were excluded.

The dataset also included demographic information (age at fracture, sex, and date of fracture) and documentation of osteoporosis (yes/no). Osteoporosis diagnosis was defined as a clinical diagnosis recorded at hospital discharge and listed among the formal discharge diagnoses in the structured EMR. Additional clinical variables included comorbidities, pre-fracture mobility status, discharge destination (home, elderly-care facility or rehabilitation ward), laboratory results, rehospitalizations, subsequent fractures, all-cause mortality, acute myocardial infarction, cerebrovascular events, and documentation of osteoporosis medications use. Documentation of osteoporosis medication use included any prescription recorded in the hospital EMR before or after the index hip fracture during the study period. Medication documentation was not time-anchored and may have reflected historical prescriptions.

The nurse practitioner’s role included identifying eligible hip-fracture admissions during hospitalization, providing ongoing inpatient clinical follow-up, reviewing discharge documentation, and facilitating inclusion of osteoporosis or osteoporotic fracture diagnoses when clinically appropriate. The nurse practitioner also coordinated inpatient rehabilitation planning and assisted in addressing intercurrent medical issues during hospitalization in collaboration with the orthopedic and medical teams. Implementation of discharge letter recommendations for further evaluation and initiation of pharmacological therapy were not within the nurse practitioner’s scope of practice.

Distributions of all relevant variables were examined in the overall cohort and stratified by the presence or absence of a documented osteoporosis diagnosis. Different analytic cohorts were used depending on data availability; these are specified in each subsection. Categorical variables were compared using Chi-square tests, while continuous variables were analyzed using independent-samples t-tests for normally distributed data and non-parametric tests (Mann–Whitney or Kruskal–Wallis, as appropriate) for non-normally distributed data.

Survival analyses were conducted using a staged analytic approach. Crude survival was described using Kaplan–Meier methods in the overall cohort hospitalized between 2007 and 2024, with between-group differences assessed using the log-rank test. Multivariable associations between documented osteoporosis diagnosis and mortality were evaluated using Cox proportional hazards regression in an exploratory analysis restricted to patients hospitalized between 2022 and 2024, corresponding to the period with available data on pre-fracture ambulatory status and complete covariate information, including age, sex, body mass index (BMI), and length of hospital stay. The proportional hazards assumption was assessed for all Cox models.

Analyses were staged according to data availability, with restricted subsets used for variables and survival analyses requiring complete covariate information.

Language editing and grammar refinement were performed with the assistance of an artificial intelligence–based tool to ensure clarity and accuracy of expression.

## Results

Our cohort included 6,933 patients. Of the study population, 4,405 patients (63.5%) were women. The mean age at the time of fracture was 81.3 years (SD ± 8.8), and the BMI was 24.6 (IQR 22.1–27.4). A subsequent osteoporotic fracture was reported in 1,714 patients (24.7%). Baseline demographic and clinical characteristics of the study population, stratified by documentation of osteoporosis diagnosis, are summarized in Table 1.

**Table 1:** Clinical characteristics of patients hospitalized for hip fracture, according to osteoporosis documentation

Characteristic	Overall (*n* = 6,933)	No osteoporosis diagnosis (*n* = 2,783)	Osteoporosis diagnosis (*n* = 4,150)	*p* value
Women, *n* (%)	4,405 (63.5)	1,717 (61.7)	2,688 (64.8)	0.010
Mean age ± SD, years	81.3 ± 8.8	79.9 ± 9.7	82.3 ± 8.0	< 0.001
BMI, median [IQR], kg/m²	24.6 [22.1–27.4]	24.5 [22.0–27.3]	24.7 [22.2–27.6]	0.031
Surgical repair, *n* (%)	6,191 (99.4)	2,203 (98.7)	3,988 (99.7)	< 0.001
Prior myocardial infarction or stroke, *n* (%)	855 (12.3)	315 (11.3)	540 (13.0)	0.115
Pre-fracture non-ambulatory status recorded, *n* (%)*		479/859 (55.8)	103/1,192 (8.6)	< 0.001
Discharge destination, *n*^ (%)				< 0.001
Home		236/616 (38.3)	506/1,861 (27.2)	
Elderly-care facility		156/616 (25.3)	561/1,861 (30.2)	
Other hospital		49/616 (7.9)	135/1,861 (7.2)	
Different ward, same hospital		175/616 (28.4)	659/1,861 (35.4)	

Baseline demographic, clinical, and outcome characteristics of 6,933 patients hospitalized for hip fracture between 2007 and 2024, stratified by documentation of osteoporosis diagnosis at discharge

^Discharge destination was available for 2,477 patients: 616 without osteoporosis diagnosis and 1,861 with osteoporosis diagnosis

Values are presented as mean ± standard deviation (SD) or median [interquartile range, IQR] for continuous variables and as number (percentage) for categorical variables. p values were derived from independent-samples t tests or Mann–Whitney U tests for continuous variables and χ² tests for categorical variables. *BMI* body mass index

**Fig. 1:**
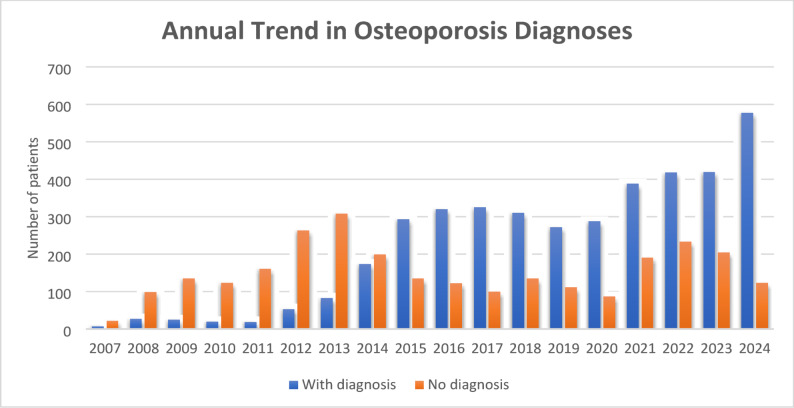
Annual trend in osteoporosis diagnosis among patients hospitalized for hip fracture between 2007 and 2024

The annual trend in osteoporosis documentation following hip fracture between 2007 and 2024 shows a distinct shift over time. During the earlier years (2007–2014), most patients (77%) did not have a documented diagnosis of osteoporosis. A marked improvement in documentation started in 2014, and from 2015 onward, the number of patients with a documented diagnosis consistently exceeded those without (Fig. 1). The highest number of documented diagnoses was recorded in 2024. Laboratory parameters, including serum calcium, phosphate, vitamin D, and creatinine levels, were comparable between groups. Median serum 25-hydroxyvitamin D levels were 20.1 [13.1–27.2] ng/mL and 20.3 [13.1–27.2] ng/mL, respectively (*p* = 0.944), and creatinine levels were also similar (median 0.89 mg/dL in both groups, *p* = 0.797).

Documentation of osteoporosis pharmacologic treatment was low and similar between the groups with and without a documented osteoporosis diagnosis (521 [12.6%] vs. 345 [12.4%], *p* = 0.84). Oral bisphosphonates were the most common agents. Among treated patients, injectable therapies were used more frequently in those with a diagnosis (114/521 [21.8%] vs. 51/345 [14.7%], *p* = 0.01).

To explore potential explanations for the discrepancy in formal osteoporosis diagnosis, we examined clinical and functional characteristics that might have influenced medical-professional decision-making, including major comorbidities (such as prior cardiovascular events), pre-fracture ambulatory status, and discharge destination.

A prior history of myocardial infarction or cerebrovascular accident was present in 13.0% of patients with a diagnosis and 11.3% of those without (*p* = 0.115).

Pre-fracture ambulatory status was available for a subset of the cohort, with consistent documentation limited to two discrete periods (2008–2012 and 2022–2024). Among patients admitted between 2022 and 2024, non-ambulatory status was uncommon and did not differ significantly between patients with and without a documented osteoporosis diagnosis (88/1,113 [7.9%] vs. 35/346 [10.1%]; *p* = 0.22).

Discharge destination data were available for 2,477 patients. Significant differences were observed between groups: patients without a documented osteoporosis diagnosis were more frequently discharged home (38.3% vs. 27.2%), while those with a diagnosis were more often discharged to an elderly care facility (30.2% vs. 25.3%) or transferred to another ward within the same hospital (35.4% vs. 28.4%) (*p* < 0.001). Discharge to another hospital was similarly uncommon in both groups.

A subsequent fracture, as recorded in the hospital EMR, occurred in 740 of 2,783 patients (26.6%) without a documented osteoporosis diagnosis and in 974 of 4,150 patients (23.5%) with a documented diagnosis (*p* = 0.003).

To further assess the impact of osteoporosis documentation, we conducted a sub-analysis restricted to patients who sustained a hip fracture between 2016 and 2024 - a period during which the involvement of the nurse practitioner in post-fracture care was well established. This subgroup included 4,751 patients, of whom 3,418 (72%) had a documented diagnosis of osteoporosis and 1,333 (28%) did not. Patients with a documented osteoporosis diagnosis were significantly older at the time of fracture, with a mean age of 82.3 years (SD 8.1), compared to 78.4 years (SD 10.3) among those without a diagnosis (*p* < 0.001). Women comprised 63.5% of the osteoporosis diagnosis group and 61.1% of the non-diagnosed group (*p* = 0.131). Nearly all patients underwent surgical treatment for the index fracture; however, this rate was higher in the osteoporosis diagnosis group (99.7% vs. 98.6%, *p* < 0.001).

In the subgroup of patients hospitalized between 2016 and 2024, crude all-cause mortality was similar in patients with and without a documented osteoporosis diagnosis (40.4% vs. 40.6%, *p* = 0.935). Unadjusted Kaplan–Meier analysis showed no significant difference in survival (log-rank *p* = 0.66).

In an exploratory multivariable Cox model restricted to 1,412 patients with complete covariate data (age, sex, BMI, length of hospital stay, and pre-fracture ambulatory status), documentation of osteoporosis was associated with lower mortality (adjusted HR 0.63, 95% CI 0.48–0.83; *p* = 0.001). This analysis was intended to assess association rather than claim causal effect.

## Discussion

In this large retrospective cohort of patients hospitalized for hip fractures, integration of a dedicated nurse practitioner into the orthopedic inpatient workflow was associated with a substantial and sustained increase in osteoporosis documentation at discharge, exceeding rates reported in many settings without formal fracture liaison. However, this improvement in documentation was not accompanied by increased recorded treatment use.

Lack of documented osteoporosis diagnosis in EMR remains a well-recognized limitation in post-fracture management [25]. Among Medicare beneficiaries in the United States, diagnosis rates remain below 30% following fragility fractures, with somewhat higher rates reported after vertebral fractures compared with hip or other fracture sites [26]. Another study found that only about 20% of patients with fragility fractures were diagnosed with osteoporosis during follow-up. Diagnosis was more frequent when the fracture was managed in an inpatient setting (27.7%) than in outpatient or community settings (9.5–15%) [27]. These inpatients were typically older and had a higher comorbidity burden, and during follow-up they sustained more subsequent fractures, reflecting their elevated baseline risk. Even among high-risk patients, osteoporosis remains substantially underdiagnosed in the absence of systematic identification measures.

Furthermore, a formal diagnosis of osteoporosis is associated with a reduced treatment gap among patients at risk for fracture [9, 28, 29]. In a large multinational European cohort of women aged ≥ 70 years, more than half were identified as being at increased fracture risk, yet over three-quarters were not receiving osteoporosis medication. Strikingly, even among those with prior spine or hip fractures, which should themselves prompt an osteoporosis diagnosis, documentation and therapy initiation remained low. When osteoporosis was formally diagnosed, however, the proportion of untreated patients declined from 94.1% to 30.9% [7]. Consistent with these observations, the GLOW study also demonstrated significantly higher use of anti-osteoporosis medication among women with a documented osteoporosis diagnosis compared with those without one [8]. 

The increased documentation rate observed in our center aligns with evidence that structured organizational interventions, such as FLS or fracture coordinators, improve secondary fracture prevention [30, 31]. Across diverse healthcare systems, nurse practitioners and coordinators embedded in post-fracture care services have consistently enhanced patient identification, increased rates of bone density testing and osteoporosis treatment, and improved adherence to follow-up [32–34].

FLS programs vary in scope, from basic patient identification to comprehensive models that include evaluation and initiation of therapy. Comprehensive models improve treatment initiation, reduce secondary fractures, and lower overall healthcare expenditures [4, 35, 36]. Their effectiveness depends on reliable patient identification, yet most rely on imperfect ICD-10 coding of fracture events. Embedding a dedicated nurse practitioner within the inpatient workflow can overcome this limitation by identifying eligible patients directly and ensuring proper documentation. Recommendations for evaluation and therapy can then be adapted to local healthcare structures and available treatments.

In many instances, post-fracture evaluation is primarily conducted in the ambulatory setting. Integrating a dedicated nurse practitioner within the inpatient workflow may help bridge this gap by ensuring early identification and standardized recommendations before discharge, while continuity of care is maintained in the community. This model allows the nurse practitioner to recommend appropriate post-discharge investigations and suggest osteoporosis therapy once the diagnostic workup is complete, enabling primary care physicians to implement treatment without delay.

A recent study proposed a computerized algorithm designed to facilitate timely initiation of osteoporosis therapy after hip fracture [37] Combining such algorithmic tools with nurse-led coordination may further enhance treatment initiation while optimizing use of specialist consultations for complex cases requiring individualized management.

In our cohort, patients with documented osteoporosis were older and more often female, though laboratory parameters and major pre-fracture comorbidities were similar between groups. Despite differences in baseline demographics and functional status, documentation of osteoporosis was not associated with significant differences in secondary clinical outcomes, including crude all-cause mortality, rehospitalizations, major infections, or cerebrovascular events.

Although differences in the proportion of recorded subsequent fractures were observed, these comparisons were not intended to estimate fracture risk. Because follow-up duration and competing mortality were not incorporated into these analyses, subsequent fractures were deliberately not modeled as time-to-event outcomes and are therefore presented as descriptive observations only. Data on clinical or radiographic vertebral fractures not requiring hospitalization were unavailable, and ascertainment of hip fractures was restricted to re-admissions to our center. While this likely resulted in underestimation of absolute fracture rates, such limitations are expected to affect both groups similarly.

Our mortality analyses were exploratory and intended to be hypothesis generating. In an adjusted analysis of a reduced recent-years cohort, documentation of osteoporosis was associated with lower mortality. However, a diagnosis alone is unlikely to directly affect survival. Missing comorbidity data preclude ruling out residual confounding. Interventional studies in which pharmacologic therapy and coordinated post-fracture care were actively implemented, including the HORIZON Recurrent Fracture Trial [38] and nurse-led FLS programs [17, 39], have reported improvements in survival. Their findings support the view that mortality benefits are more plausibly driven by coordinated care and effective treatment rather than diagnostic documentation alone.

Despite the substantial increase in osteoporosis documentation, recorded pharmacologic treatment in the hospital EMR remained low. Interpretation of treatment rates is limited by incomplete capture of outpatient prescriptions and adherence, as most patients were followed in community clinics not linked to our database. Medication data were restricted to hospital-affiliated visits and readmissions, precluding assessment of whether documentation influenced post-discharge management.

Differences in documentation patterns may reflect workflow factors or clinical prioritization, although detailed ward-level decision-making data were unavailable. BMD results were largely missing because DXA testing is typically performed in community settings and not routinely integrated into hospital records. Socioeconomic status and insurance coverage were not assessed; however, the study was conducted within a universal public healthcare system in which access to care is standardized. As a single-center retrospective EMR-based analysis, residual confounding and incomplete system capture cannot be excluded.

## Conclusions

The major strengths of this study include its large sample size, extended follow-up duration, and consistent inpatient management by a single nurse practitioner, minimizing inter-provider variability. To our knowledge, this is one of the largest single-center analyses evaluating the impact of a targeted nurse-led intervention on osteoporosis documentation following hip fracture.

In conclusion, integration of a dedicated nurse practitioner into the orthopedic inpatient workflow was associated with a marked improvement in osteoporosis documentation after hip fracture. This focused, resource-efficient model may serve as a feasible alternative to comprehensive FLS in hospitals with limited infrastructure, facilitating systematic recognition and documentation of osteoporosis risk in patients hospitalized with hip fracture.

## Supplementary Information

Supplementary Material 1.

## Data Availability

The datasets generated and analyzed during the current study were obtained using the MDClone platform at Sheba Medical Center. Due to institutional and national data-protection regulations, the raw datasets cannot be publicly shared. Aggregated data are available from the corresponding author upon reasonable request and subject to institutional approval.

## Abbreviations

AI: Artificial intelligence
BMD: Bone mineral density
BMI: Body mass index
CI: Confidence interval
DXA: Dual–energy X–ray absorptiometry
EMR: Electronic medical record
FLS: Fracture Liaison Service
HR: Hazard ratio
ICD: International Classification of Diseases
IQR: Interquartile range
IRB: Institutional Review Board
IV: Intravenous
MDClone: Medical Data Clone (synthetic data generation platform)
SD: Standard deviation
SC: Subcutaneous
SMC: Sheba Medical Center
