# Supplementary material for: Osteoporosis documentation following hip fracture: a retrospective cohort study from a tertiary hospital
Source: BMC Musculoskelet Disord. 2026 Mar 23;27:365. doi: 10.1186/s12891-026-09745-6 (PMC13130409; doi:10.1186/s12891-026-09745-6)
Supplement: Supplementary file 1 — Supplementary Material 1. [file 12891_2026_9745_MOESM1_ESM.docx]

**Supplement A** - **List of hip fractures included in the query:**

This supplement details the diagnostic terms used for cohort identification and their corresponding ICD-9-CM and ICD-10-CM codes. Multiple textual variants referring to the same anatomical fracture location were mapped to unified ICD categories for analysis.

| **Original clinical term** | **ICD-9-CM** | **ICD-10-CM** |  |
| --- | --- | --- | --- |
| Closed fracture of base of neck of femur [femoral] | 820.03 | S72.04X- |  |
| Closed fracture of hip | 820.9 | S72.9X- |  |
| Closed fracture of neck of femur [femoral] | 820.00 | S72.00X- |  |
| Closed fracture proximal femur, subtrochanteric [femoral] | 820.22 | S72.2X- |  |
| Closed intertrochanteric fracture [femur, femoral] | 820.21 | S72.14X- |  |
| Closed pertrochanteric fracture [femur, femoral] | 820.20 | S72.10X- |  |
| Femoral neck fracture garden 1-2 | 820.02 | S72.03X- |  |
| Femoral neck fracture garden 3-4 | 820.03 | S72.03X- |  |
| Femoral neck fracture, base of | 820.03 | S72.04X- |  |
| Fracture of base of neck of femur closed | 820.03 | S72.04X- |  |
| Fracture of base of neck of femur, closed | 820.03 | S72.04X- |  |
| Fracture of femor intertrochanteric | 820.21 | S72.14X- |  |
| Fracture of femor neck closed, nos | 820.00 | S72.00X- |  |
| Fracture of femural neck | 820.00 | S72.00X- |  |
| Fracture of greater trochanter [femur, femoral] | 820.8 | S72.8X- |  |
| Fracture of head of femur [femoral, hip] | 820.09 | S72.01X- |  |
| Fracture of intertrochanteric femur | 820.21 | S72.14X- |  |
| Fracture of neck of femur – closed | 820.00 | S72.00X- |  |
| Fracture of neck of femur [femoral, hip] | 820.00 | S72.00X- |  |
| Fracture of neck of femur \| garden stage 1-2 [femoral, hip] | 820.02 | S72.03X- |  |
| Fracture of neck of femur \| garden stage 1-2 [femoral] | 820.02 | S72.03X- |  |
| Fracture of neck of femur \| garden stage 3-4 [femoral, hip] | 820.03 | S72.03X- |  |
| Fracture of neck of femur \| garden stage 3-4 [femoral] | 820.03 | S72.03X- |  |
| Fracture of neck of femur \| nonunion [femoral, hip] | 733.82 | S72.0XXK |  |
| Fracture of proximal end of femur [femoral, hip] | 820.9 | S72.9X- |  |
| Fracture of unspecified part of neck of femur, closed | 820.00 | S72.00X- |  |
| Fracture of unspecified part of neck of femur, open | 820.10 | S72.00X- |  |
| Fx base femoral nck-clos | 820.03 | S72.04X- |  |
| Fx neck of femur nos-cl | 820.00 | S72.00X- |  |
| Hip fracture | 820.9 | S72.9X- |  |
| Hip replacement, partial, following fracture 271250 | - | - |  |
| Intertrochanteric fracture [femur, femoral] | 820.21 | S72.14X- |  |
| Intertrochanteric fx-cl | 820.21 | S72.14X- |  |
| Neck of femur fracture | 820.00 | S72.00X- |  |
| Neck of femur fracture (closed) | 820.00 | S72.00X- |  |
| Neck of femur fracture (open) | 820.10 | S72.00X- |  |
| Non union femoral neck fracture | 733.82 | S72.0XXK |  |
| Pertrochanteric fracture [femur, femoral] | 820.20 | S72.10X- |  |
| Pertrochanteric fracture \| a1 [femur, femoral] | 820.20 | S72.11X- |  |
| Pertrochanteric fracture \| a2 [femur, femoral] | 820.20 | S72.12X- |  |
| Pertrochanteric fracture \| a3 [femur, femoral] | 820.20 | S72.13X- |  |
| Pertrochanteric fracture a1 | 820.20 | S72.11X- |  |
| Pertrochanteric fracture a2 | 820.20 | S72.12X- |  |
| Pertrochanteric fracture a3 | 820.20 | S72.13X- |  |
| Pertrochanteric fracture of femur | 820.20 | S72.10X- |  |
| Pertrochanteric fracture of femur closed | 820.20 | S72.10X- |  |
| Pertrochanteric fracture of femur, closed | 820.20 | S72.10X- |  |
| Pertrochanteric fx-clos | 820.20 | S72.10X- |  |
| Subcapital fracture of neck of femur | 820.02 | S72.03X- |  |
| Subcapital fracture of neck of femur [femoral, hip] | 820.02 | S72.03X- |  |
| Subcapital fracture of neck of femur [femoral] | 820.02 | S72.03X- |  |
| Trochanteric fx nos-clos | 820.8 | S72.8X- |  |

**Footnote**

Garden classification, AO/OTA subtypes (A1–A3), laterality, displacement, and encounter-type/healing extensions in ICD-10-CM were collapsed to the category level for cohort construction. Diagnosis inclusion was based on structured electronic medical record documentation rather than billing codes alone.
